# Maternal Risk Factors Associated With Neonatal Outcome in Primiparous, Machala, Ecuador

**DOI:** 10.7759/cureus.33860

**Published:** 2023-01-17

**Authors:** Tania Diciana Arevalo Cordova, Brigida Maritza Agudo Gonzabay, Jorge Armando García Maldonado, Carlos Hugo Medina Quizhpe, Nathaly Lisseth León León

**Affiliations:** 1 Medicine/Medical Science Career, Technical University of Machala, Machala, ECU; 2 Obstetrics and Gynecology/Medical Science Career, Technical University of Machala, Machala, ECU

**Keywords:** newborn, outcome neonatal, associated factors, maternal, risk factors

## Abstract

Introduction

The resulting neonatal, weight of the newborn (NB) is considered as a health indicator since the nutritional status of the neonate can have repercussions on the growth and development of the child until adulthood. Secondly, preterm delivery is associated with several maternal risk factors, such as the presence of anemia, adolescence, or advanced age. The aim of the study was to determine the maternal risk factors related to neonatal outcomes in primiparous.

Methods

A descriptive, observational, quantitative, longitudinal, and non-experimental study was conducted. Data were collected from women who gave birth from September 2021 to August 2022, in a Microsoft Excel database and the analysis was performed using SPSS software, version 26.

Results

The study population consisted of 224 pregnant women, aged 16 to 41 years, with a mean of 21 years (SD ± 4 years), the most predominant age range was under 20 years, with 53.33%, 81.7% were of middle socioeconomic status, 50.4% had basic education, 89.7% self-identified as mestizo race, 86.2% were of Ecuadorian nationality, and 96.0% resided in the urban area. A total of 97.8% were term NB, 69.9% were normal weight, and 96.4% had an Apgar score of 8 to 10 in the first minute after birth. Maternal factors related to Apgar 7 were adolescent and elderly women, with an odds ratio (OR) of 2.180; having maternal comorbidity OR: 2.0612; the factors related to preterm and post-term neonates were the degree of primary and basic education, OR: 2.0, without statistical significance (p>0.05). And in relation to low weight and high weight, we have an academic education OR: 3.0417, without statistical significance (p>0.05); and mothers with a history of previous abortions, OR: 8.6000, with high statistical significance (p<0.05).

Conclusions

Among the main maternal factors related to neonatal outcome in primiparous pregnant women were educational level, age, number of prenatal checkups, and history of previous abortions.

## Introduction

Globally, in 2019, 2,400,000 infants died in their first month of life, and 6700 neonates die every day, with a decrease recorded since 1990. The majority of all neonatal deaths (75%) occur during the first week of life, and approximately one million newborns (NB) die in the first 24 hours of life. Preterm births, birth-related complications, and infections accounted for the majority of NB deaths [1].

Among the main maternal risk factors associated with neonatal outcomes are high age, prolonged pregnancy, and excessive weight gain during pregnancy [2]. In addition, there are maternal factors related to diseases during gestation, such as hypertensive disorders during pregnancy, which can cause intrauterine growth restriction and increased neonatal mortality in the fetus [3].

Regarding the resulting neonatal outcome, the weight of the NB is considered a health indicator, since the nutritional status of the neonate can have repercussions on the growth and development of the child to adulthood [4]. On the other hand, the gestational age of the neonate is considered a physiological determinant for extrauterine adaptation, and this is an important milestone for survival prognosis, mainly in the early neonatal period [5,6]. Prematurity exposes children from birth to malnutrition, delayed development, and growth, which can ultimately hinder learning and normal functions during childhood, adolescence, and adulthood [7].

In the study conducted by Genes [8], heredity and preeclampsia were identified as maternal factors associated with an increased risk of preterm delivery. Calderón et al. [9], in their study, established premature rupture of membranes, cervico-vaginitis, urinary tract infection, and anemia as maternal risk factors for preterm delivery; while Rodríguez et al. [10] identified maternal age over 35 years (15%), placenta previa (9%), and urinary tract infections (46%) together with premature rupture of membranes and cervicovaginitis as maternal risks of prematurity.

Preterm delivery is associated with several maternal risk factors, such as the presence of anemia, adolescent or advanced age (aged primigravidae), high levels of catecholamines in urine during gestation, consumption of licit or illicit drugs, premature rupture of membranes, arterial hypertension and preeclampsia, transvaginal bleeding, urinary tract infections, oligohydramnios, history of abortion [11].

Pregnant patients with hypertensive disorders have a greater propensity for their NB to suffer complications that are evident at birth. There are risk factors that affect the development of the embryo and fetus: extreme maternal age (risk of preeclampsia), poor prenatal control, and concomitant maternal diseases [12]. In view of the above, the objective of this research is to determine the maternal risk factors related to neonatal outcomes in primiparous at the Velasco Ibarra Health Center, 2021-2022.

## Materials and methods

A descriptive, quantitative, longitudinal, study was carried out. Statistical and analytical method was used. The study was conducted on pregnant women who attended the Type C "Velasco Ibarra" Health Center, in the city of Machala, El Oro Province, Ecuador, from September 1, 2021, to August 31, 2022, who met the inclusion criteria.

Inclusion criteria

Primiparous pregnant, who attended the "Velasco Ibarra" Health Center in the active phase of labor, from September 1, 2021, to August 31, 2022.

Exclusion criteria

Multigestational pregnant, multiparous, who attended the "Velasco Ibarra" Health Center during the active phase of labor from September 1, 2021, to August 31, 2022, or did not meet the inclusion criteria.

The required information was collected directly from the medical records of each pregnant woman and NB and from the delivery matrix of the Delivery and Recovery Unit, referring to maternal age, level of education, ultrasound scans performed, maternal morbidities, the sex of the NB, gestational age, weight, Apgar, and neonatal co-morbidity.

Gestational age was recorded as gestational age, whether the NB was a term or preterm, considering the classification of the World Health Organization, in preterm NB, less than 37 weeks; term NB, 37-41 weeks and post-term NB, 42 weeks or more [13,14].

Quantitative analysis of the information was then performed using Statistical Product and Service Solutions (SPSS) (IBM SPSS Statistics for Windows, Version 26.0, Armonk, NY). The results were presented in tables and graphs made in Microsoft Excel 2016 for subsequent interpretation. For the association of variables, the odds ratio was used, with a confidence index of 95%, for which the variables were dichotomized and 2 x 2 tables were used.

Permission was obtained from the Director of the Health Center to collect the information, taking care of the confidentiality of the information, without revealing the personal data of the patients in this study.

## Results

The study population consisted of 224 pregnant women, aged 16 to 41 years, with a mean of 21 SD ± 4 years. The most predominant age range was under 20 years of age, 81.7% were of middle socioeconomic status, 50.4% had basic education, 89.7% self-identified as mestizo, 86.2% were of Ecuadorian nationality, and 96.0% resided in the urban area (Table 1).

**Table 1:** Sociodemographic data n = 224

Variable	No. (%)
Age (years)	21 ± 4
Age by range	
≤ 20 years	126 (56.3)
21-30 years	90 (40.2)
31-40 years	7 (3.1)
≥ 41 years	1 (0.4)
Socioeconomic status	
Low	41 (18.3)
Medium	183 (81.7)
High	0 (0.0)
Educational level	
Less than secondary school	150 (66,9)
Baccalaureate	54 (24.1)
University	20 (8.9)
Self-identification ethnic	
Afrodescendant	16 (7.14)
Mulatto	4 (1.8)
Montubia	1 (0.4)
Mestiza	201 (89.7)
Caucasian	1 (0.4)
Other ethnicities	7 (3.1)
Nationality	
Ecuadorian	193 (86.2)
Spanish	1 (0.4)
Peruvian	3 (1.3)
Venezuelan	27 (12.1)
Residence	
Urban	215 (96.0)
Rural	9 (4.0)

Regarding the neonatal outcome, 53.1% of the NB were female, 97.8% were at term, 69.9% were normal weight, and 96.4% had an Apgar of 8 to 10 in the first minute after birth (Table 2).

**Table 2:** Neonatal outcome n = 224

Variable	No. (%)
Gender	
Male	105 (46.9)
Female	119 (53.1)
Gestational age	
Preterm	3 (1.3)
Term	219 (97.8)
Post-term	2 (0.9)
Weight	
Underweight	4 (1.8)
Normal weight	217 (96.9)
High weight	3 (1.3)
Apgar 1st minute	
1-4	1 (0.4)
5-7	7 (3.1)
8-10	216 (96.4)

In reference to the maternal factors associated with neonatal outcome, the Apgar 7 at the first minute of birth was found in 75% of mothers with primary or basic education and in adolescents or older women, with an odds ratio (OR) of 2.00 and 2.1840 respectively. The prematurity and postmaturity were present in five neonates, 80% of whom were born to mothers with primary or basic education, OR: 2.00; 40% of mothers were elderly or adolescents OR: 0.4651; 100% of mothers had had ≤ 2 prenatal checkups OR: 0.6627; regarding NB with low birth weight and high birth weight, 85.7% occurred in mothers with primary or basic education, OR: 3.0417; 71.4% were the product of adolescent or elderly pregnancy, OR: 1.8056, 100% had less than two controls OR: 0.9124; There are no low or high weight neonates, mothers with morbidities O: 0.8710, all with p>0.05; and 42.9% of the mothers had a history of previous abortions OR: 8.6000, p<0.05 (Table 3).

**Table 3:** Maternal factors associated with neonatal outcome * p<0.05 n = 224

Variable	Neonatal Outcome		Odds Ratio (OR)		
	No. (%)	No. (%)	OR	IC: 95%	p
	Apgar ≤ 7	Apgar ≥ 8			
Educational level					
Elementary school	6 (75.0)	144 (66.7)	0.2500	0.0454 to 1.3757	0.1111
Baccalaureate and University	2 (25.0)	72 (33.3)			
Age (years)					
≤ 20 o ≥ 35 years	6 (75.0)	125 (57.9)	2.1840	0.4309 to 11.0687	0.3455
21-34 years	2 (25.0)	91 (42.1)			
Prenatal Checkups					
≤ 2	7 (87.5)	205 (94.9)	0.3756	0.0424 to 3.3273	0.3789
≥ 3	1 (12.5)	11 (5.1)			
Maternal Comorbidity					
Yes	1 (14.3)	14 (6.5)	2.0612	0.2367 a 17.9486	0.5124
No	7 (100.0)	202 (93.1)			
Previous Abortions					
Yes	2 (25.0)	46 (21.3)	1.2319	0.2406 to 6.3072	0.8024
No	6 (75.0)	170 (78.7)			
	Pre and Post Term	Term			
Educational level					
Elementary school	4 (80.0)	146 (66.7)	2.0000	0.2196 to 18.2184	0.5386
Baccalaureate and University	1 (20.0)	73 (33.3)			
Age (years)					
≤ 20 o ≥ 35 years	2 (40.0)	129 (58.9)	0.4651	0.0762 to 2.8403	0.4070
21-34 years	3 (60.0)	90 (41.1)			
Prenatal Checkups					
≤ 2	5 (100.0)	207 (94.5)	0.6627	0.0347 to 12.6713	0.7846
≥ 3	0 (0.0)	12 (5.5)			
Maternal Comorbidity					
Yes	0 (0.0)	15 (6.9)	1.1994	0.0634 to 22.7061	0.9035
No	5 (71.4)	204 (93.1)			
Previous Abortions					
Yes	0 (0.0)	48 (21.9)	0.4114	0.0224 to 7.5660	0.5500
No	5 (100.0)	171 (79,1)			
	Low and high weight	Normal weight			
Educational level					
Elementary school	6 (85.7)	144 (66.4)	3.0417	0.3594 to 25.7413	0.3073
Baccalaureate and University	1 (14.3)	73 (33.6)			
Age (years)					
≤ 20 o ≥ 35 years	5 (71.4)	126 (58.1)	1.8056	0.3427 to 9.5138	0.4859
21-34 years	2 (28.6)	91 (41.9)			
Prenatal Checkups					
≤ 2	7 (100.0)	205 (94.5)	0.9124	0.0493 to 16.9029	0.9509
≥ 3	0 (0.0)	12 (5.5)			
Maternal Comorbidity					
Yes	0 (0.0)	15 (6.9)	0.8710	0.0475 to 15.9721	0.9258
No	7 (100.0)	202 (93.1)			
Previous Abortions					
Yes	3 (42.9)	45 (20.7)	8.6000	1.7586 to 42.0566	0.0079*
No	4 (57.1)	172 (79.3)			

## Discussion

There was only one primiparous aged 41 years, representing 0.40%, and the most predominant age range was under 20 years of age, in agreement with the study conducted by Agudo et al. [15], in which the age with the highest number of pregnant was 20 years, with 11.67%, followed by 19 years, with 10.12%. Similarly, in the study conducted by Arévalo et al. [16], during pregnancy, the age with the highest prevalence was 20 years, with 10.8%.

There was a high percentage of adolescent pregnancies and a small number of older pregnant women. In contrast to the study conducted by Islas et al. [17]. In Mexico, where 2.6% of mothers were adolescents, while 56.6% corresponded to the group of older pregnant (over 35 years of age). On the other hand, in the study carried out in primiparous in an Amazonian hospital in Peru, 73.71% of the pregnancies were older, 58.25% had primary and secondary education, and 57.22% lived in rural areas [18].

In general, in the present study, the neonatal outcome was within normal parameters, with few neonates with low Apgar, prematurity or postmaturity, low weight or higher weight. In concordance with the study conducted at the Hospital Hipótilo Unaune, Peru, in which the male sex prevailed in the neonates, with 51.57%; 93.7% had a term gestational age, 71.133% had adequate weight [18]. In the study of neonatal outcomes between vertical and horizontal delivery, carried out between April 2018 and May 2019, by Agudo et al. [15], 99.68% of NB were at term and 93.10% had normal weight.

Regarding the maternal factors associated with neonatal outcome, is evident an association between Apgar ≤ 7 with adolescent pregnancy and maternal morbidity. Data were concordant with the study conducted by Chambilla-Coila et al. [18], where the Apgar less than 7 was related to maternal age older than 35 years, primary education, lack of prenatal controls, and maternal morbidity.

A relationship was found among pregnant women with basic levels of study (primary and basic education), with the postmaturity and prematurity of the NB. Data were similar to those found in the study by Castillo et al. [19], where maternal factors related to prematurity were found to be adolescent or elderly maternal age, low economic level, maternal history of abortion, inadequate prenatal control, and maternal comorbidities.

It is evident that there is a relationship between low or high weight, the educational background of the mother, as well as having a history of previous abortions. Betancourt et al. [20] state that prenatal care helps to significantly reduce maternal and neonatal morbidity and mortality, premature births, and the number of low birth weight babies, improving the quality of life of pregnant women and their children in the long term.

## Conclusions

The study population consisted of 224 pregnant women, aged 16 to 41 years, with a mean age of 21 years (SD ± 4 years), the most predominant age range was under 20 years, with medium socioeconomic status, basic education, Ecuadorian nationality, and residing in the urban area. Maternal factors related to the resulting premature or postmature neonatal were the low educational level of the mother. There is an association between the history of previous abortions, with the resulting neonatal, low or high weight, with high statistical significance.

Adequate prenatal follow-up is recommended in pregnant primiparous, focused on identifying maternal factors associated with unfavorable neonatal outcomes, in order to implement the prevention and health promotion strategies necessary to reduce the number of low-weight NB, and neonatal complications.
